# Case Report: Diagnostic value of spectral CT in primary adrenal lymphoma

**DOI:** 10.3389/fonc.2026.1639755

**Published:** 2026-03-26

**Authors:** Xiang Zhuang, Xi xi Jin, Li wen Feng, Hui Zhang

**Affiliations:** Department of Radiology, Baotou Cancer Hospital, Baotou, China

**Keywords:** adrenal gland, dual-energy CT, iodine density, lymphoma, spectral CT

## Abstract

Primary adrenal lymphoma (PAL) is a rare extranodal malignant tumor that poses significant diagnostic challenges due to its nonspecific clinical and imaging features. We report a case of unilateral PAL in a 55-year-old female presenting with flank pain and a hypodense adrenal mass. The lesion was characterized using dual-layer spectral detector computed tomography (CT) with analyses including virtual monoenergetic imaging, iodine density mapping, effective atomic number (Z-eff) calculation, and spectral attenuation curve evaluation. The mass demonstrated low attenuation, reduced iodine concentration (0.38–0.69 mg/mL), low Z-eff values (7.51–7.71), and a flat spectral curve pattern—findings consistent with hypovascular lymphoid tissue. Subsequent 18F-FDG PET/CT confirmed intense focal metabolic activity in the adrenal lesion (SUV_max 14.3). Biopsy confirmed the diagnosis of diffuse large B-cell lymphoma (DLBCL), non-germinal center B-cell (non-GCB) subtype. Spectral CT facilitated early non-invasive diagnosis, enhanced lesion conspicuity, and guided appropriate biopsy. This case highlights its diagnostic utility. The present case demonstrates the potential diagnostic value of multi-parameter spectral CT in differentiating PAL from other adrenal masses. These preliminary findings suggest the possible clinical utility of this technique; however, its role requires further validation through large-scale studies in the future.

## Introduction

Primary adrenal lymphoma (PAL) accounts for less than 1% of all non-Hodgkin lymphomas and constitutes under 0.9% of all malignant tumors (1). The disease typically arises primarily within the adrenal glands without lymph node involvement and is more commonly observed in elderly male patients, often presenting with bilateral involvement. Non-specific symptoms such as abdominal pain, fatigue, and loss of appetite frequently lead to diagnostic delays. While conventional CT and MRI offer limited specificity, spectral (dual-energy) CT provides more detailed tissue characterization through quantitative imaging techniques (2).

## Case Report

A 55-year-old female patient (BMI = 26.7) presented with a two-week history of left-sided abdominal pain accompanied by mild abdominal distension and decreased appetite. No systemic symptoms or lymphadenopathy were observed. An ultrasound examination revealed a 3.5×3.7×4.5 cm mass in the left adrenal gland.

Image Acquisition and Analysis: Spectral CT examination was performed using a Philips IQon Spectral CT 128-slice scanner. Scan parameters were as follows: tube voltage, 120 kVp; tube current, automated mAs modulation; pitch, 0.969; detector collimation, 64 × 0.625 mm; rotation time, 0.4 s/rotation. Image reconstruction and spectral analysis were performed on a Philips IntelliSpace Portal workstation (version 12.0) using its built-in spectral analysis toolkit. For quantitative analysis, Regions of Interest (ROIs) were independently placed and measured by two radiologists, each with over five years of experience in abdominal imaging. Standardized ROI placement rules were followed:

ROIs were carefully placed to avoid areas of visible necrosis, calcification, and prominent vessels.

Circular or oval ROIs with a fixed area of 15–20 mm² were placed within the solid portion of the adrenal lesion and, as specifically noted for region S2, within the “involved adrenal branch” to ensure consistency and comparability.

Each observer performed three repeated measurements in the same region, and the average value was recorded as the final spectral CT parameter for that region, including iodine concentration (IC, mg/mL), CT number at 40 keV, and the spectral curve.

Laboratory Investigations: Relevant endocrine laboratory tests were performed. Catecholamines and their metabolites, serum cortisol rhythm and adrenocorticotropic hormone (ACTH) levels, the renin-aldosterone ratio, and electrolytes (including serum potassium and sodium) were all within normal reference ranges.

**Figure 1:**
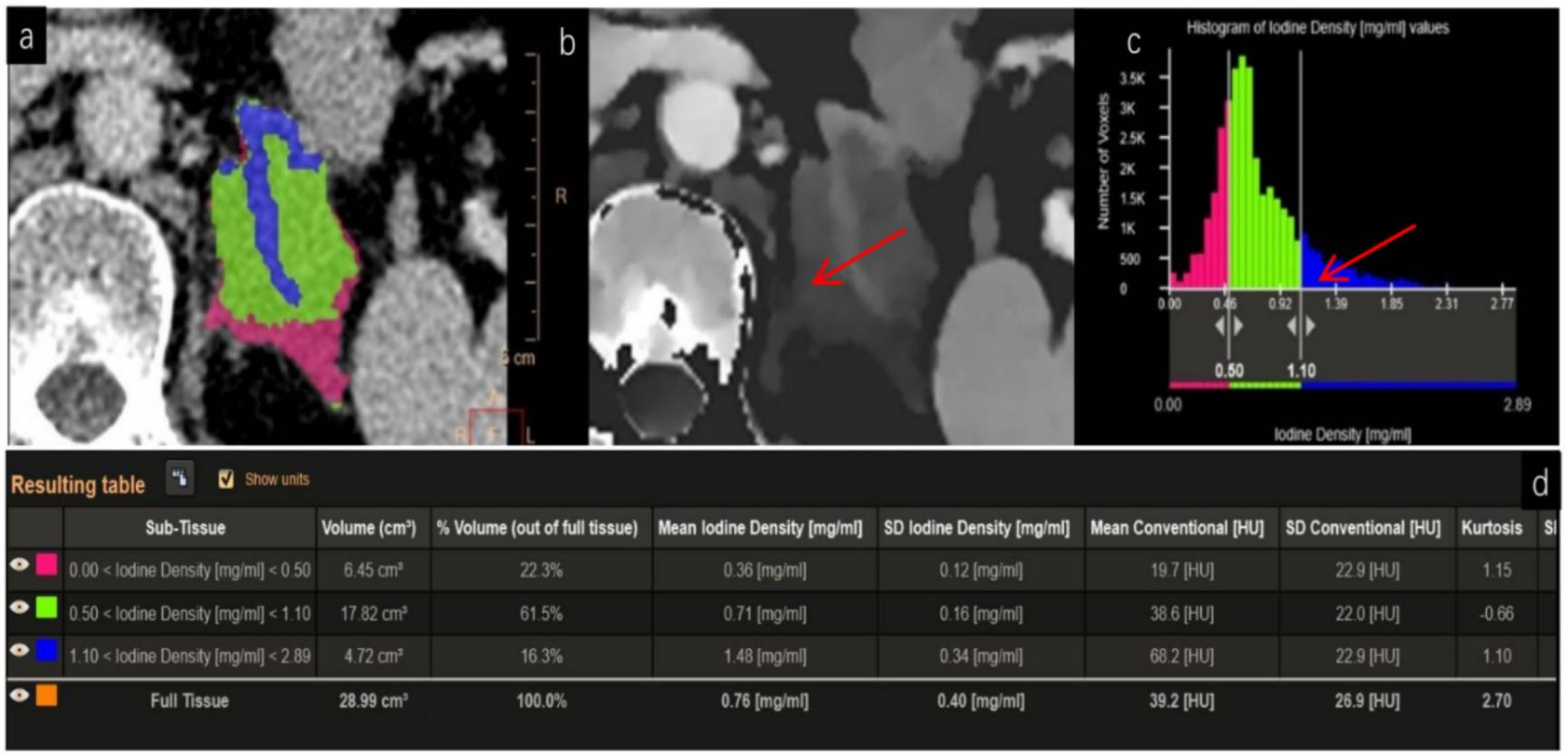
Iodine-based tumor and adrenal tissue volumetric analysis. **(a)** Three-dimensional (3D) volume rendering: Blue color represents normal adrenal tissue; red and green indicate PAL lesions with low and moderate iodine concentrations, respectively. **(b)** Corresponding iodine density map: The PAL lesion, marked by a red arrow, continues to show reduced iodine uptake (0.38–0.69 mg/mL), consistent with the findings in **Figure 2**. **(c)** Segmented histogram of the iodine density map: Blue corresponds to normal adrenal tissue, while red and green represent lesion areas with low and moderate iodine concentrations, respectively. A red arrow highlights the normal adrenal tissue region. **(d)** Frequency histogram illustrating tissue volume distribution across varying iodine density levels: The lesion demonstrates heterogeneous perfusion. The volume of adrenal tissue (highlighted in blue) measures 4.72 mL, accounting for 16.3% of the total analyzed tissue volume. Clinical significance: Tissue segmentation and quantitative analysis based on the volume of interest (VOI) provide objective data that can support precise treatment planning, such as delineating surgical resection margins. VOI, volume of interest; PAL, primary adrenal lymphoma.

**Figure 2:**
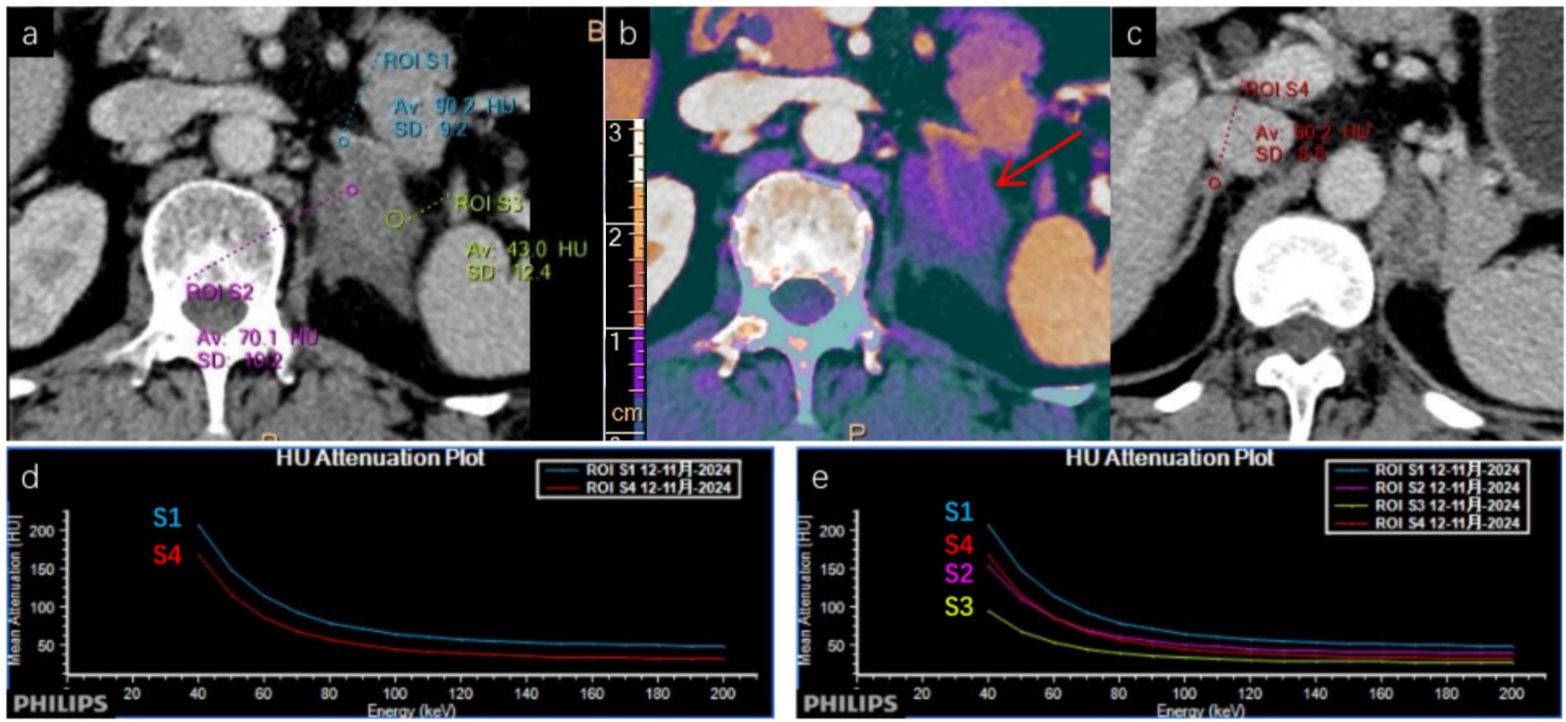
Analysis of spectral attenuation curves. **(a, c)** Conventional CT images with regions of interest (ROIs) delineated: S1 (normal left adrenal gland), S2 (partially involved adrenal branch), S3 (PAL lesion, indicated by red arrow), and S4 (normal right adrenal gland). Quantitative attenuation values: The lesion (S3) demonstrated low density, with Hounsfield unit (HU) values ranging from 26.1 to 38.5, while the adjacent normal adrenal tissue (S1/S4) showed an HU value of 75.4. **(b)** Iodine density map: Red arrows highlight residual normal adrenal tissue and the PAL lesion. The lesion exhibited reduced iodine uptake (0.38–0.69 mg/mL), compared to 1.82 mg/mL in the normal adrenal tissue (S1/S4). **(d, e)** Spectral attenuation curves for ROIs S1–S4: S1 and S4 displayed parallel curves, indicating homogeneous composition of the normal adrenal tissue. S2 showed a curve shift, suggesting partial infiltration by lymphoma. S3 presented a flat curve with low slope, a characteristic feature of lymphoma tissue. Key finding: Compared to conventional CT, spectral CT images clearly visualize the spatial relationship among normal adrenal tissue (S1, S4), residual adrenal tissue (S2), and the PAL lesion (S3), enabling precise delineation of tumor boundaries from surrounding tissue. CT, computed tomography; PAL, primary adrenal lymphoma; HU, Hounsfield units; ROI, region of interest.

On conventional CT, the lesion demonstrated homogeneous low attenuation (26.1–38.5 HU) relative to the adjacent adrenal tissue (75.4 HU). At 40 keV virtual monoenergetic imaging, the lesion’s conspicuity improved significantly (59.7–84.0 HU vs. 187.3 HU). Iodine density within the lesion was markedly reduced (0.38–0.69 mg/mL) as shown in **Figure 1** compared to normal adrenal tissue (1.82 mg/mL). The effective atomic number (Z-eff) values in the lesion (7.51–7.71) were lower than those in normal adrenal tissue (8.3), findings suggestive of hypovascular, lymphoid tissue. Spectral curves derived from four regions of interest—normal adrenal glands (S1, S4), the tumor (S3), and an involved adrenal branch (S2)—confirmed the differences in attenuation characteristics as shown in **Figure 2**, supporting the diagnosis of lymphoma.

**Figure 3:**
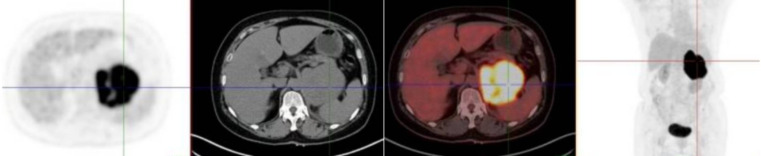
PET-CT image. Coronal PET-CT demonstrates significant FDG uptake in the left adrenal mass (SUVmax 14.3), with no evidence of distant metastasis.

**Figure 4:**
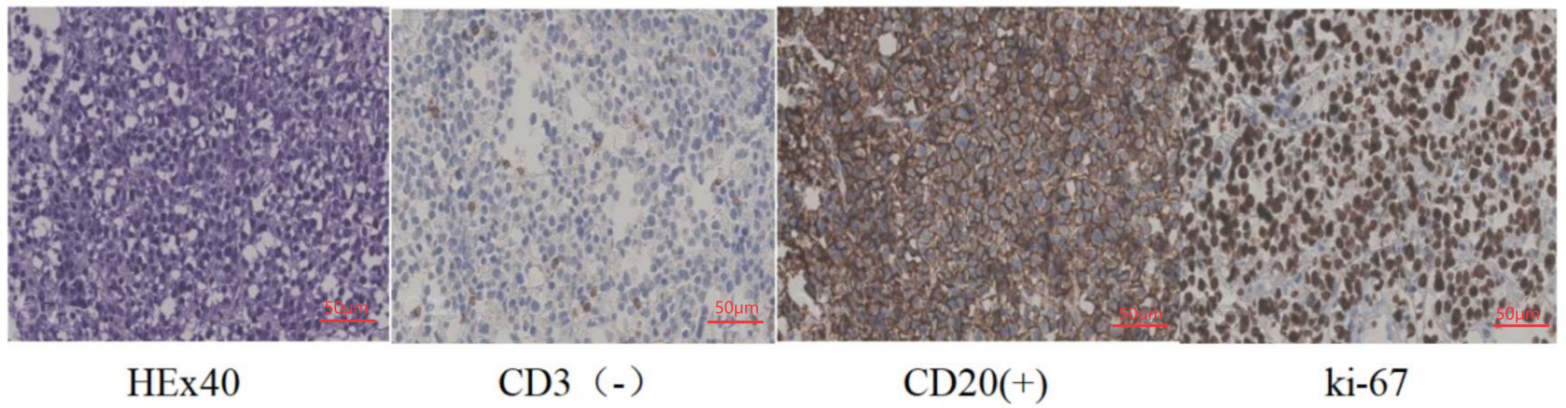
Histopathology and immunohistochemistry. Microscopic sections demonstrate diffuse large B-cell lymphoma with a high Ki-67 index (approximately 90%). Immunohistochemical staining was positive for Bcl-2, CD19, CD20, and CD79a/b. c-Myc expression was observed in approximately 40% of cells. Tests for BCL2/c-MYC rearrangement and EBV-EBER were negative.

Subsequent 18F-FDG PET-CT revealed a hypermetabolic left adrenal mass (SUVmax 14.3) with no evidence of extra-adrenal uptake as shown in **Figure 3**. An ultrasound-guided biopsy confirmed the diagnosis of diffuse large B-cell lymphoma (DLBCL), non-germinal center B-cell (non-GCB) subtype. Immunohistochemistry as shown in **Figure 4** was positive for Bcl-2, CD19, CD20, CD79a/b, and Ki-67 (approximately 90%). c-Myc expression was observed in about 40% of cells, while tests for BCL2/c-MYC rearrangement and EBV-EBER were negative.

## Discussion

Non-Hodgkin lymphoma accounts for approximately 90% of all lymphomas, while PAL is relatively rare, comprising about 3% of extranodal lymphomas (3). Primary adrenal lymphoma (PAL) is strongly associated with Epstein-Barr virus (EBV) infection in the majority of cases, typically indicated by positive EBER *in situ* hybridization. The oncogenic role of EBV in lymphomagenesis—mediated through latent proteins such as LMP1 and EBNA2, which activate signaling pathways including NF-κB and JAK/STAT—has been well established (4). However, both the World Health Organization (WHO) classification and several published studies acknowledge the existence of rare EBV-negative PAL subtypes. The pathogenesis of these variants may involve alternative oncogenic pathways independent of EBV or differences in the host immune microenvironment. The present case represents one such EBV-negative variant, underscoring that even when EBV testing is negative, a diagnosis of PAL should still be considered when strongly supported by morphological and immunophenotypic features. Imaging plays a crucial role in distinguishing PAL from other adrenal lesions (5). While ^18^F-FDG PET-CT demonstrates high sensitivity and specificity for detecting extranodal involvement, its routine application is limited by cost, relatively low spatial resolution, radiation exposure, and the need for radiotracers (6–8). Spectral CT offers several key advantages in this context: (a)Low-keV monoenergetic imaging (e.g., 40 keV):Enhances iodine contrast, improving the delineation between hypodense lymphoma tissue and normal adrenal parenchyma. (b)Iodine density mapping: Enables quantification of perfusion. The significantly reduced iodine concentration within PAL (0.38–0.69 mg/mL) reflects its hypovascular nature and homogeneous cellular density, consistent with previous reports. (c) Effective atomic number (Z-eff) mapping: Identifies elemental composition. The lower Z-eff values of lymphoma tissue (7.5–7.7) compared to normal adrenal tissue (8.3) help define tumor margins and suggest a distinct elemental profile. (d) Spectral attenuation curve analysis: Characterizes energy-dependent attenuation. The flat, low-slope curve of PAL differs from that of residual normal tissue and other adrenal pathologies.

Characteristic spectral CT manifestations of PAL include: (a) Unilateral or bilateral hypodense adrenal masses. (b) Mild, progressive, homogeneous enhancement (12–14). (c) Infiltrative margins encasing adjacent structures (15). (d) Due to the sparse stromal components and limited small vessels within the stroma of adrenal lymphoma, PAL is typically hypovascular, resulting in low iodine uptake and reduced Z-eff values. (e) The histopathological characteristics of PAL account for its flat, low-slope spectral curve, primarily due to the paucity of cellular stroma, which results in relatively homogeneous X-ray attenuation properties. (f) Clearly delineate the tumor margins and their relationship with adjacent organs, such as the intact renal capsule (16).

Adrenal lymphoma is characterized by densely packed tumor cells with scant cytoplasm and extremely low intracellular lipid content. Its primary constituents are proteins, nucleic acids, and water—components richer in elements with relatively higher atomic numbers, such as oxygen, nitrogen, and phosphorus. Consequently, its theoretical Z-eff value is expected to be closer to or slightly higher than that of soft tissue (e.g., 8.3–8.8). In the present case, however, the measured Z-eff was 7.6, which falls below this expected range. While this finding appears contradictory to the assumption that lymphoma should exhibit a higher Z-eff, it provides a valuable starting point for deeper pathophysiological exploration. Several factors in the differential diagnosis warrant consideration: Tumor compositional heterogeneity: Despite the overall high cellularity of lymphoma, the presence of necrosis, edema, or sparse myxoid stroma within the lesion—regions rich in water (H_2_O, containing low-atomic-number hydrogen)—could lower the overall measured Z-eff. Selection of the region of interest (ROI):Even when carefully avoiding macroscopically visible necrotic areas, the ROI may still include tumor stroma with relatively lower cellular density, influencing the average value. Specific features of PAL: The unique biology of primary adrenal lymphoma may contribute to this discrepancy. This apparent contradiction underscores the limitation of relying on a single parameter, including Z-eff, for differentiation. A comprehensive interpretation requires integrating Z-eff with iodine concentration (IC), spectral curve morphology, and conventional CT features such as enhancement degree and homogeneity.

Although the case did not undergo detailed histopathological subtyping, its imaging features—moderate-to-low vascularity and significant post-treatment reduction—combined with a clear response to standard lymphoma chemotherapy (R-CHOP regimen), suggest it may belong to a chemotherapy-sensitive subtype. Literature indicates that within diffuse large B-cell lymphoma, the non-GCB subtype may be associated with more aggressive clinical behavior, yet it can still exhibit a high response rate to first-line chemotherapy (17). Post-treatment follow-up imaging at an external hospital showed a marked reduction in lesion size from approximately 3.5 × 3.7 × 4.5 cm to 3.4 × 1.8 cm, supporting the lesion’s sensitivity to systemic chemotherapy. This also underscores the critical importance of accurate diagnosis in initiating the correct treatment strategy. Future standardized subtyping of PAL will contribute to a deeper understanding of the relationship between its imaging manifestations and prognosis.

Primary adrenal lymphoma often demonstrates moderate to low-level enhancement on imaging, which may be attributed to its unique pathophysiological basis. Lymphoma is typically characterized by a diffuse and homogeneous proliferation of tumor cells, differing from the abundant tumor angiogenesis commonly observed in carcinomas. Its blood supply relies more on the pre-existing vascular framework of the infiltrated tissue, with relatively insignificant neovascularization. Additionally, adrenal lymphoma contains scant stromal components with only a small number of microvessels, which hinders the rapid penetration of contrast agent into the tumor mass. This explains its frequent manifestation as intermediate to low enhancement on contrast-enhanced CT and in iodine concentration measurements. The relatively low iodine concentration measured by spectral CT (approximately 0.38–0.69 mg/mL in the venous phase in this case) serves as a quantitative reflection of these microscopic pathophysiological features on macroscopic imaging.

The relatively low iodine concentration and effective atomic number observed on spectral CT in this case not only reflect the tumor’s cellular composition and vascular characteristics but may also indicate a distinct tumor metabolic phenotype. The mechanistic target of rapamycin (mTOR) signaling pathway, particularly mTOR complex 1 (mTORC1), serves as a central hub integrating signals from growth factors, nutrients, and energy. It plays a pivotal role in regulating cell growth, metabolism, and protein synthesis. Its aberrant activation is closely linked to the progression of various tumors, including lymphoma (18, 19). Notably, recent research further reveals that the activity state of mTORC1 profoundly influences cellular transcriptional programs and proteostasis (20, 21), which directly determines a tumor’s metabolic activity, proliferation rate, and even cellular composition. Based on this, we propose an exploratory hypothesis: The activity state of the mTOR signaling pathway in PAL may influence its quantitative imaging phenotype on spectral CT by shaping its level of metabolic activity, protein synthesis rate, and cellular density. For instance, active mTORC1 signaling, supporting higher metabolic demands, might theoretically correspond to different iodine uptake kinetics (affecting iodine concentration) and cellular component profiles (affecting the effective atomic number). Although no study has yet directly established a link between the mTOR pathway and spectral CT parameters in PAL, this theoretical framework provides a novel biological perspective for interpreting the “dual-low” imaging pattern (low iodine concentration, low effective atomic number) observed in our case. It suggests that future research could focus on exploring the correlation between the molecular metabolic characteristics of tumors (such as mTOR pathway-related markers) and their quantitative phenotypic expression on advanced imaging techniques like spectral CT. This approach may lead to the development of imaging biomarkers capable of non-invasively reflecting the intrinsic biological behavior of tumors, thereby enabling more precise diagnosis and treatment response assessment.

Spectral CT also plays a crucial role in differentiating other adrenal masses (As shown in **Table 1**): (a) For instance, adenomas typically exhibit intracellular lipid content, resulting in lower iodine concentration; Adrenal adenomas lack lipids rich in low-atomic-number elements such as carbon and hydrogen; therefore, there is no major factor to lower the overall effective atomic number (Z-eff), which is typically relatively high. However, the average slopes of the spectral curves differ among various adenomas, which may be a combined result of varying ratios of lipids mixed with other substances within the voxels (22). (b) Pheochromocytomas often exhibit necrosis and cystic changes, are typically hypervascular, and therefore demonstrate features such as hypervascularity, cystic alterations, and a steep descending spectral slope (23). (c) Metastases exhibit varied imaging appearances: hypervascular metastases (e.g., from renal cell carcinoma) typically show elevated iodine concentration/Z−eff values, whereas hypovascular metastases (e.g., from colorectal or lung cancer) generally demonstrate lower values (2).; Metastases often arise in the adrenal medulla, which has higher water content and lower lipid content, therefore resulting in a “descending” pattern on spectral curves.

**Table 1:** Spectral CT parameters of different adrenal lesions: comparison with primary adrenal lymphoma (PAL) case.

Spectral parameter	Adrenal adenoma (9)	Adrenal metastasis (10)	Pheochromocytoma (11)	PAL (Present case)
Iodine Density (vp)	1.75 ± 0.93 mg/mL	1.16 ± 0.55 mg/mL	2.47 ± 2.20 mg/mL	0.38–0.69 mg/mL
Effective Atomic Number	8.24 ± 0.42	7.95 ± 0.29	8.38 ± 0.53	7.51–7.71
Spectral Curve Pattern	Spoon, Rising, Declining	Steep Decline (Low Slope)	Decline (High Slope)	Decline (Low Slope)

Hypervascular adrenal metastases, such as those originating from renal cell carcinoma, typically demonstrate high iodine uptake. Using a dual-source CT system, Martin and colleagues evaluated iodine density in adrenal lesions (23). Their study reported an iodine density of approximately 3.2 ± 1.4 mg/mL in metastases during the portal venous phase. The authors suggested that this finding might be attributed to underlying histopathological variations, noting that most metastases in their cohort originated from primary renal cell carcinomas—tumors known for their hypervascularity and potential endogenous lipid content—which could explain the discrepancy compared to other studies. In contrast, Nagayama et al. (2) reported that in the portal venous phase, the iodine density in adenomas (2.4 ± 0.8 mg/mL) was significantly higher than in metastases (1.7 ± 0.5 mg/mL) (p < 0.001). They attributed this finding to variability in contrast agent kinetics and iodine uptake among different lesions. Similarly, Wu et al. (10) demonstrated that the venous-phase iodine density was higher in adenomas than in metastases (1.75 ± 0.93 mg/mL vs. 1.16 ± 0.55 mg/mL, p < 0.05). It is plausible that the adrenal metastases analyzed in these latter studies were primarily derived from hypovascular primary tumors, such as those commonly originating from lung cancer.

## Treatment and follow-up

**Figure 5:**
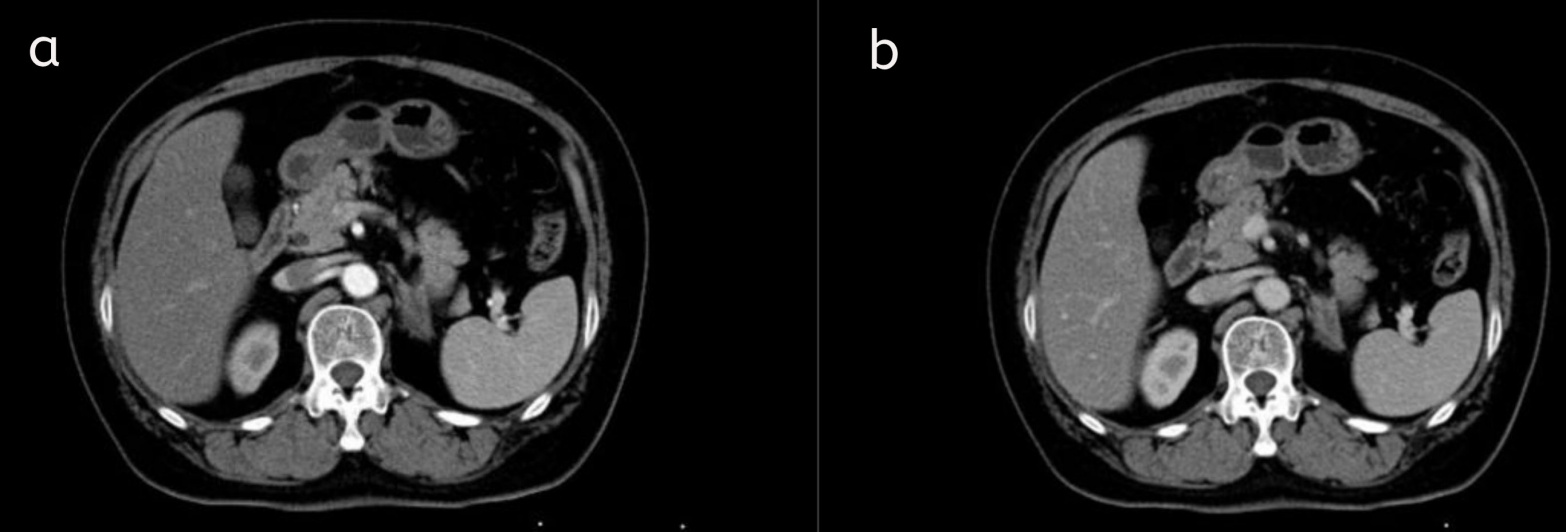
Post-treatment follow-up CT images: arterial phase **(a)** and venous phase **(b)**. Following standard lymphoma therapy with the R-CHOP regimen, follow-up abdominal CT demonstrated a reduction in the size of the left adrenal lymphoma lesion (decreased to approximately 3.4 cm × 1.8 cm) compared to pre-treatment imaging. The mass effect of the original lesion was alleviated, and its degree of enhancement was reduced. These imaging findings are consistent with a positive treatment response.

Based on the imaging and pathological (biopsy) findings, the patient was definitively diagnosed with primary adrenal lymphoma (diffuse large B-cell lymphoma). Following discussion by a multidisciplinary team, the patient promptly commenced standard first-line lymphoma therapy with the R-CHOP regimen, consisting of: zanubrutinib (160 mg, day 1), rituximab (600 mg, day 1), pirarubicin (70 mg, day 1), cyclophosphamide (1.2 g, day 1), vincristine (2 mg, day 1), and prednisone (60 mg, days 1–5). After treatment initiation, the patient underwent regular clinical assessments. Following completion of the initial treatment cycles, a follow-up examination was performed at an external hospital on December 25, 2025 as shown in **Figure 5**. Post-treatment non-contrast and contrast-enhanced adrenal CT scans revealed a significant reduction in the size of the original left adrenal mass. The lesion decreased from approximately 3.5 × 3.7 × 4.5 cm to approximately 3.4 × 1.8 cm (maximum cross-sectional measurement). This imaging change indicates a favorable response to the chemotherapy regimen. As of the writing of this report, the patient continues to receive subsequent treatment cycles and is under regular follow-up. The patient has tolerated the treatment well overall, with no reports of severe complications.

Early and accurate non-invasive diagnosis of PAL is crucial, as its treatment approach (chemotherapy, immunotherapy, radiotherapy) differs significantly from that of adenoma or pheochromocytoma, which typically require surgical intervention. Spectral CT can improve diagnostic specificity, guide targeted biopsy, and potentially reduce unnecessary interventions.

## Conclusion

To our knowledge, no prior studies have systematically applied multi-parameter spectral CT—specifically integrating multiple quantitative tools such as 40-keV monoenergetic images, iodine concentration maps, effective atomic number (Z-eff) maps, and spectral attenuation curves—to the diagnosis and differential diagnosis of adrenal PAL. Therefore, this case report may be regarded as the first to provide such a comprehensive multi-parameter spectral CT characterization of this rare tumor, offering a novel evaluation model and representing an initial report of its kind.

Spectral CT enhances the non-invasive assessment of adrenal lesions by providing comprehensive tissue characterization. In this case, the technique facilitated the visualization, differential diagnosis, and histopathological correlation of primary adrenal lymphoma (PAL). Incorporating spectral CT into the diagnostic workflow may improve diagnostic accuracy and timeliness, thereby enabling more personalized management of adrenal masses. However, as a single-center retrospective case study, these findings are preliminary. The generalizability and diagnostic performance of this approach require further validation through prospective, large-scale, multicenter studies.

## Data Availability

The original contributions presented in the study are included in the article/supplementary material, further inquiries can be directed to the corresponding author/s.
